# Supplementary material for: Optimal inter-electrode distances for maximizing single unit yield per electrode in neural recordings
Source: Microsyst Nanoeng. 2026 Jan 26;12:41. doi: 10.1038/s41378-025-01115-x (PMC12835126; doi:10.1038/s41378-025-01115-x)
Supplement: Supplementary file 1 — Supplementary Information [file 41378_2025_1115_MOESM1_ESM.pdf]

Supplementary Information

Optimal inter-electrode distances for maximizing single unit yield per electrode in neural recordings

Domokos Meszéna[1,2,3], Ward Fadel[1,2], Robert Tóth[4,5], Angelique C. Paulk[3], Sydney S. Cash[3], Ziv Williams[6], Tamás Kiss[4], Marcell Stippinger[4], Lucia Wittner[1,7], Richárd Fiáth[1,2,+,*], and Zoltán Somogyvári[4,8,+]

[1]HUN-REN Research Centre for Natural Sciences, Institute of Cognitive Neuroscience and Psychology, Integrative Neuroscience Group, Budapest, H-1117, Hungary
[2]Pázmány Péter Catholic University, Faculty of Information Technology and Bionics, Budapest, H-1083, Hungary
[3]Center for Neurotechnology and Neurorecovery, Department of Neurology, Massachusetts General Hospital, Harvard Medical School, Boston, MA 02114, USA
[4]HUN-REN Wigner Research Center for Physics, Institute for Particle and Nuclear Physics, Department of Computational Sciences, Theoretical Neuroscience and Complex Systems Research Group, Budapest, H-1121, Hungary
[5]University of Oxford, Nuffield Department of Clinical Neurosciences, Oxford, OX1 3TH, UK
[6]Department of Neurosurgery, Massachusetts General Hospital, Harvard Medical School, Boston, MA 02114, USA
[7]Semmelweis University, Faculty of Medicine, Department of Neurosurgery and Neurointervention, Budapest, H-1145, Hungary
[8]Axoncord LLC., Budapest, Hungary
[*]Correspondence: fiath.richard@ttk.hu
[+]these authors contributed equally to this work

Recording ID	Animal ID	Target	AP	ML	DV
1	Rat1	Neocortex (S1HL)	-2.00	1.80	2.20
2	Rat1	Thalamus (LDVL, AV, VA, VL)	-2.00	1.80	6.20
3	Rat1	Thalamus (LDVL, Po, VPM)	-2.85	2.36	6.00
4	Rat2	Neocortex (S1Tr)	-2.70	2.51	2.20
5	Rat2	Thalamus (LDVL, Po, VPM)	-2.70	2.51	5.70
6	Rat3	Thalamus (LDVL, Po, VPM)	-2.70	2.65	5.60
7	Rat3	Thalamus (LDVL, Po, VPM, VPL)	-2.70	2.65	7.20
8	Rat4	Neocortex (PtA)	-3.45	2.42	2.10
9	Rat5	Neocortex (S1Tr)	-2.80	2.10	1.80
10	Rat6	Neocortex (S1Tr)	-2.90	2.22	1.80
11	Rat6	Neocortex (PtA)	-3.60	2.22	1.80

Supplementary Table 1. Stereotaxic coordinates of targeted brain regions in rats. AP, anteroposterior; ML, mediolateral; DV, dorsoventral; S1HL, hindlimb region of the primary somatosensory cortex; S1Tr, trunk region of the primary somatosensory cortex; PtA, parietal association cortex; LDVL, ventrolateral part of the laterodorsal nucleus; Po, posterior nucleus; VPM, ventral posteromedial nucleus; VPL, ventral posterolateral nucleus; AV, anteroventral nucleus; VA, ventral anterior nucleus; VL, ventrolateral nucleus.

Recording ID	Animal ID	Target	AP	ML	DV
1	Mouse1	S1Tr	-1.60	1.50	1.50
2	Mouse2	S1Tr	-1.60	1.50	1.50
3	Mouse3	S1Tr	-1.60	1.57	1.80
4	Mouse4	S1Tr	-1.60	1.51	1.40
5	Mouse5	S1Tr	-1.60	1.51	1.30
6	Mouse6	S1Tr	-1.50	1.59	1.30
7	Mouse7	S1Tr	-1.90	1.63	1.40

Supplementary Table 2. Stereotaxic coordinates of targeted cortical areas in mice. AP, anteroposterior; ML, mediolateral; DV, dorsoventral; S1Tr, trunk region of the primary somatosensory cortex.

Dataset	256 ch	128 ch	64 ch	32 ch	16 ch
Rat neocortex (KS2)	106.50 ± 52.14	60.00 ± 14.64	51.67 ± 22.76	29.50 ± 7.89	12.17 ± 6.75
Rat neocortex (KS1)	59.83 ± 14.80	43.17 ± 9.37	32.50 ± 8.48	18.50 ± 5.61	7.33 ± 3.20
Rat neocortex (MS4)	69.67 ± 23.64	54.33 ± 18.28	44.67 ± 17.10	24.67 ± 8.98	8.17 ± 4.02
Rat neocortex (SC)	56.33 ± 21.38	40.00 ± 17.89	35.00 ± 11.87	25.33 ± 9.31	7.67 ± 2.88
Rat thalamus (KS2)	92.00 ± 19.60	67.00 ± 19.13	45.00 ± 11.05	31.20 ± 16.96	11.80 ± 6.87
Mouse neocortex (KS2)	61.14 ± 20.10	39.14 ± 14.57	27.57 ± 12.59	12.00 ± 6.22	7.00 ± 4.40

Supplementary Table 3. Single unit yields across different rodent datasets, recordings and spike sorting algorithms (average ± standard deviation). ch, channel; KS1, Kilosort1; KS2, Kilosort2; MS4, MountainSort4; SC, SpyKING CIRCUS.

**Dataset**	**192 ch**<br>n = 2	**96 ch**<br>n = 4	**64 ch**<br>n = 6	**48 ch**<br>n = 8	**38 ch**<br>n = 10	**19 ch**<br>n = 20
Human neocortex	79.50 ± 16.26	62.00 ± 19.44	39.33 ± 8.09	36.38 ± 7.01	34.40 ± 10.09	11.00 ± 4.48

Supplementary Table 4. Single unit yields in the human dataset (average ± standard deviation). The total number of data files generated for each downsampled recording with different channel numbers is also shown. Kilosort2 was used for spike sorting. ch, channel.

Dataset	256 ch	128 ch	64 ch	32 ch	16 ch	Total
Rat neocortex (KS2)	639	360	310	177	73	1559
Rat neocortex (KS1)	359	259	195	111	44	968
Rat neocortex (MS4)	418	326	268	148	49	1209
Rat neocortex (SC)	338	240	210	152	46	986
Rat thalamus (KS2)	460	335	225	156	59	1235
Mouse neocortex (KS2)	428	274	193	84	49	1028

Supplementary Table 5. Total number of single units across different rodent datasets, recordings and spike sorting algorithms. ch, channel, KS1, Kilosort1; KS2, Kilosort2; MS4, MountainSort4; SC2, SpyKING CIRCUS.

Dataset	192 ch<br>n = 2	96 ch<br>n = 4	64 ch<br>n = 6	48 ch<br>n = 8	38 ch<br>n = 10	19 ch<br>n = 20	Total
Human neocortex	159	248	236	291	344	220	1498

Supplementary Table 6. Total number of single units in the human dataset. The total number of data files generated for each downsampled dataset with different channel numbers is also shown. Kilosort2 was used for spike sorting. ch, channel.

Dataset	256 ch	128 ch	64 ch	32 ch	16 ch
Rat neocortex (IN)	15.36 ± 6.16	15.89 ± 6.63	13.87 ± 6.62	16.09 ± 10.97	12.92 ± 12.06
Rat neocortex (PC)	84.64 ± 6.16	84.11 ± 6.63	86.13 ± 6.62	83.91 ± 10.97	87.08 ± 12.06
Mouse neocortex (IN)	24.23 ± 7.36	26.83 ± 10.11	26.10 ± 12.01	28.31 ± 17.81	24.81 ± 17.84
Mouse neocortex (PC)	75.77 ± 7.36	73.17 ± 10.11	73.90 ± 12.01	71.69 ± 17.81	75.19 ± 17.84

Supplementary Table 7. Proportion (in %) of putative interneurons (IN) and principal cells (PC) across neocortical rodent datasets and recordings (average ± standard deviation). ch, channel.

**Dataset**	**192 ch**<br>n = 2	**96 ch**<br>n = 4	**64 ch**<br>n = 6	**48 ch**<br>n = 8	**38 ch**<br>n = 10	**19 ch**<br>n = 20
Human neocortex (IN)	27.92 ± 24.93	22.60 ± 13.18	18.61 ± 10.23	22.71 ± 14.41	20.70 ± 11.70	29.42 ± 15.13
Human neocortex (PC)	72.08 ± 24.93	77.40 ± 13.18	81.39 ± 10.23	77.29 ± 14.41	79.30 ± 11.70	70.58 ± 15.13

Supplementary Table 8. Proportion (in %) of putative interneurons (IN) and principal cells (PC) across human neocortical recordings (average ± standard deviation). The total number of data files generated for each downsampled dataset with different channel numbers is also shown. ch, channel.

Dataset	p-value	Statistical test
Rat neocortex (IN)	0.994	Kruskal-Wallis
Rat neocortex (PC)	0.994	Kruskal-Wallis
Mouse neocortex (IN)	0.995	Kruskal-Wallis
Mouse neocortex (PC)	0.994	Kruskal-Wallis
Human neocortex (IN)	0.453	Kruskal-Wallis
Human neocortex (PC)	0.453	Kruskal-Wallis

Supplementary Table 9. Statistical comparisons of interneuron (IN) and principal cell (PC) ratios across different inter-electrode distances.

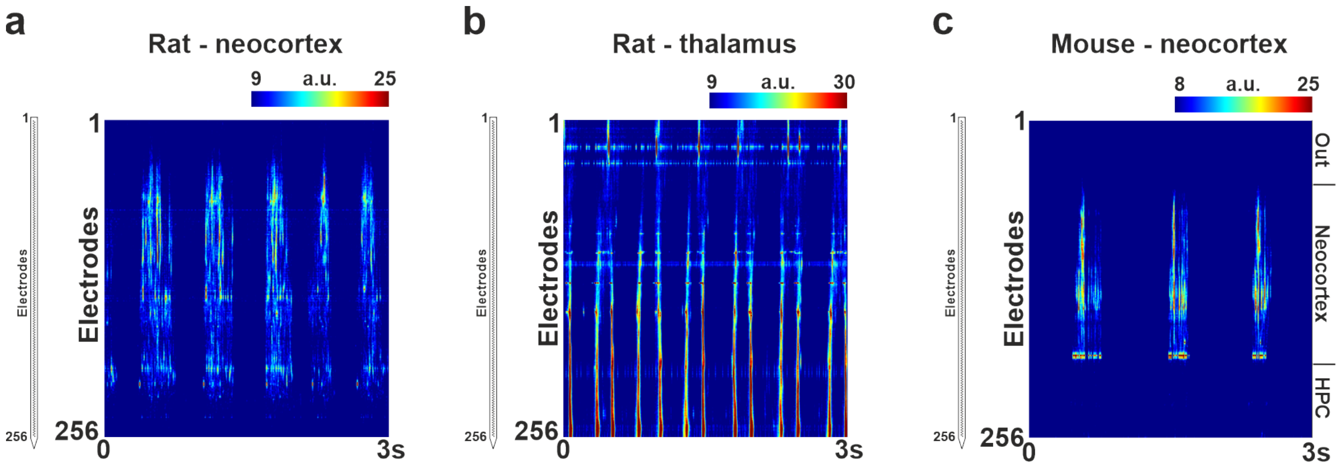

Supplementary Figure 1. Representative 3-second-long examples of high-density neuronal recordings (500–5000 Hz frequency band) obtained from the rat neocortex (**a**), rat thalamus (**b**) and mouse neocortex (**c**). On the left, the schematic of the section of the probe shank containing the microelectrodes (small black squares) is displayed. On the right, color maps visualizing spiking activity across all channels (i.e., depth profiles) are presented. For these depth profiles, before plotting, data on each channel was rectified, then smoothed with a 50 Hz low-pass filter (third-order Butterworth filter). Warmer colors on the color maps indicate higher spiking activity. Rat and mouse data were acquired under ketamine/xylazine anesthesia. HPC, hippocampus.

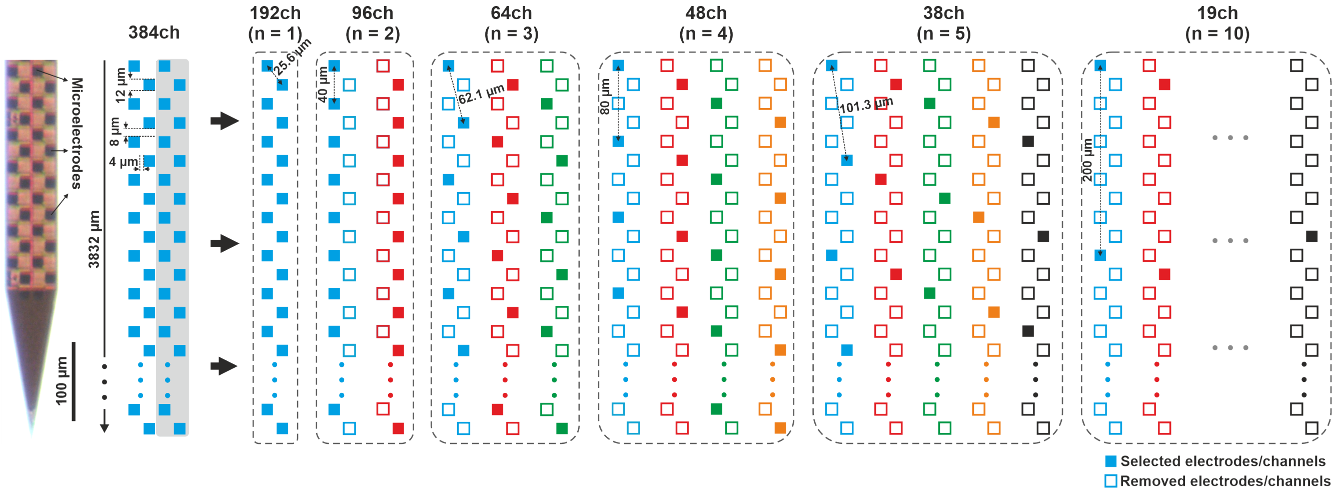

Supplementary Figure 2. Spatial downsampling of high-density Neuropixels recordings. Lower channel (ch) count recordings, corresponding to reduced spatial resolutions, were generated from the original 384-channel human cortical recordings (spatial resolution of recordings decreases from left to right). Only data recorded by the rightmost two columns of electrodes (gray shaded area) were used for analysis. To increase the sample size, all possible electrode configurations were generated for each spatial resolution (channel count). Different configurations are indicated with different colors. The size of the microelectrodes, along with the inter-electrode distances for both the original and downsampled recordings, are displayed. A stereomicroscopic image of the tip region of the Neuropixels probe used for human recordings is shown on the left.

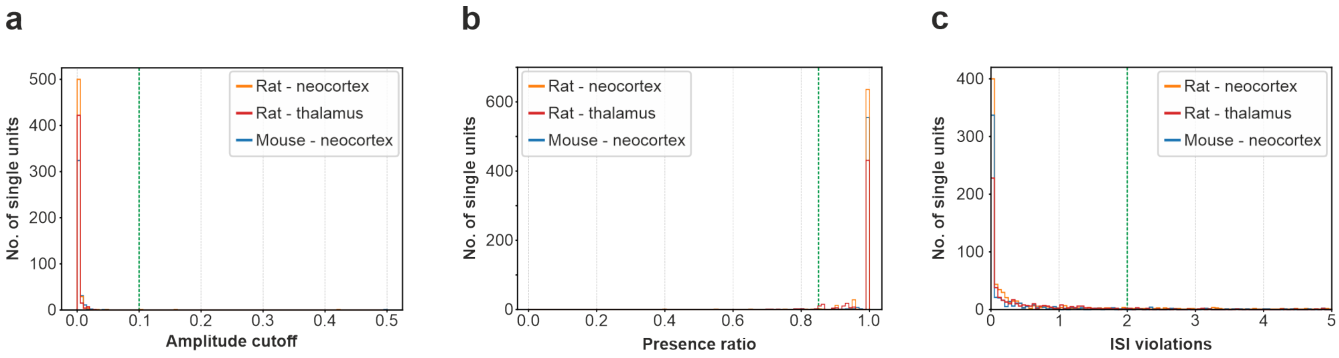

Supplementary Figure 3. Distribution of different quality metrics for the rat and mouse datasets. (**a**) Amplitude cutoff, (**b**) presence ratio, (**c**) interspike-interval (ISI) violations. The vertical dashed green lines indicate the thresholds used to exclude low-quality single units.

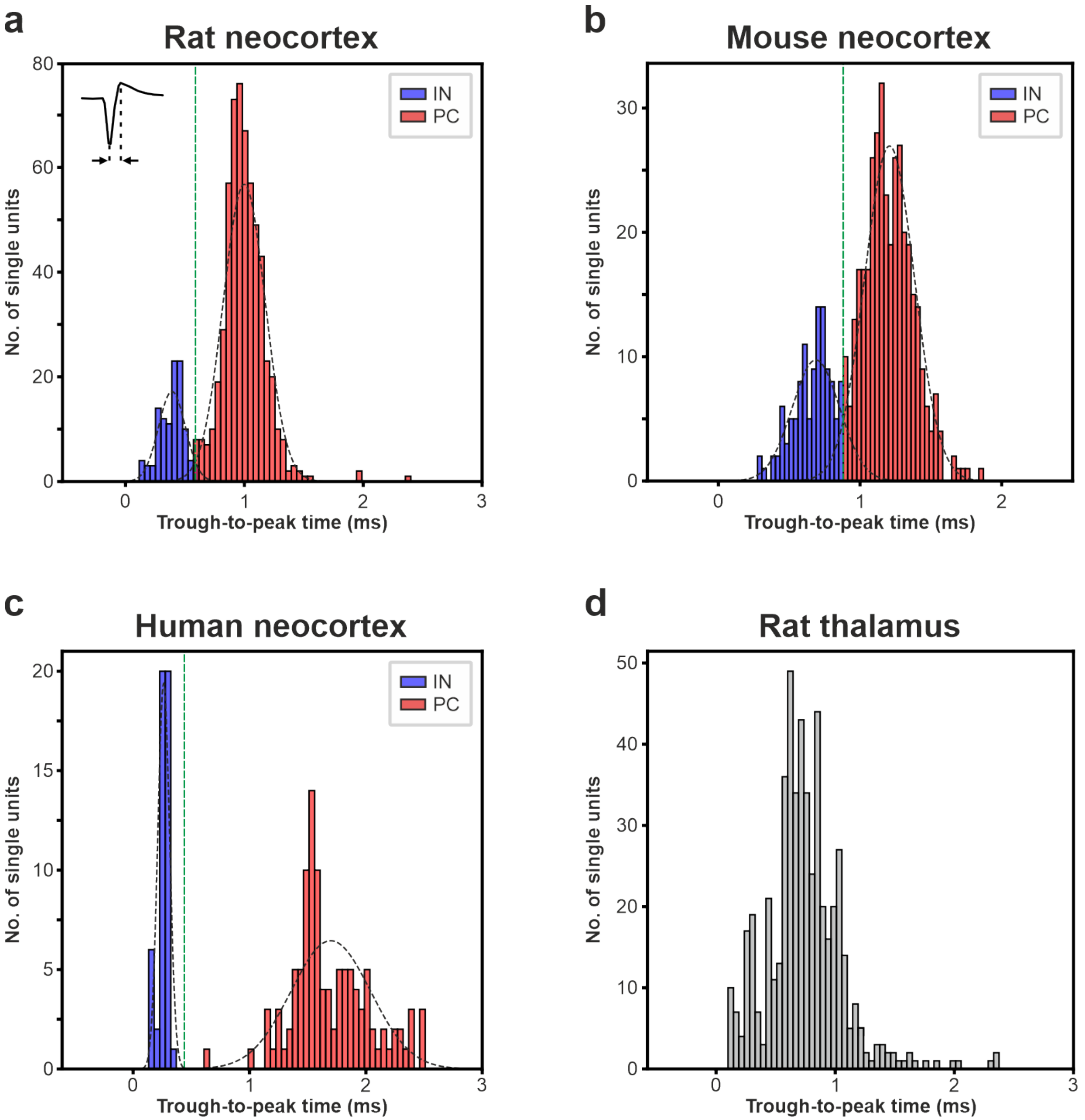

Supplementary Figure 4. Distribution of spike durations (trough-to-peak or peak-to-valley time, see inset in panel (**a**) for all datasets. (**a**) Rat neocortex, (**b**) mouse neocortex, (**c**) human neocortex, (**d**) rat thalamus. Single units were classified as putative interneurons (IN, blue) or principal cells (PC, red). Gaussian curves (dashed black curves) were fitted to the histograms to separate the two neuron populations. The separation threshold is indicated by a vertical dashed green line (separation thresholds: rat neocortex, 0.585 ms; mouse neocortex, 0.880 ms; human neocortex, 0.347 ms). No bimodal distribution was observed in the rat thalamus dataset; therefore, neurons were not separated in this case.

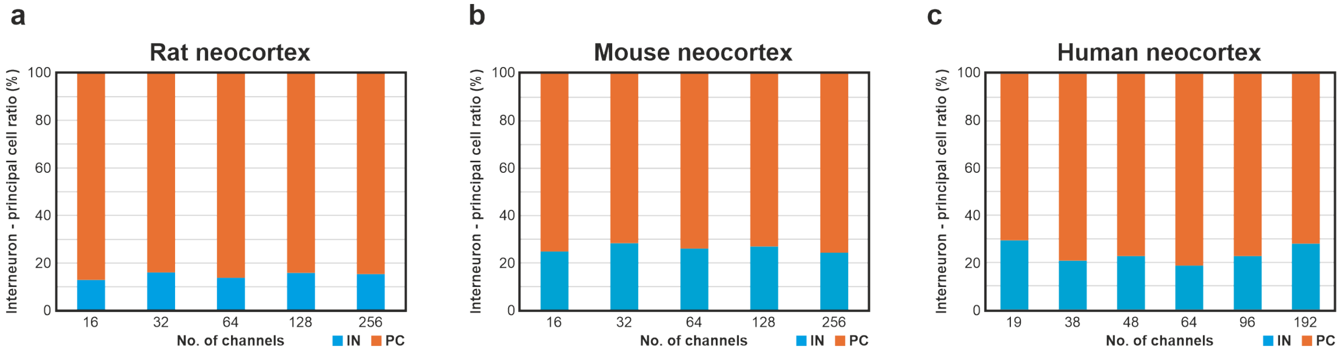

Supplementary Figure 5. Proportion of putative inhibitory interneurons (IN, blue) and excitatory principal cells (PC, red) in the neocortex of rats (**a**), mice (**b**) and humans (**c**) across recordings with different channel numbers. Note that, for a particular species, the ratio of the two neuron types remains closely the same compared between recordings with different spatial resolutions.

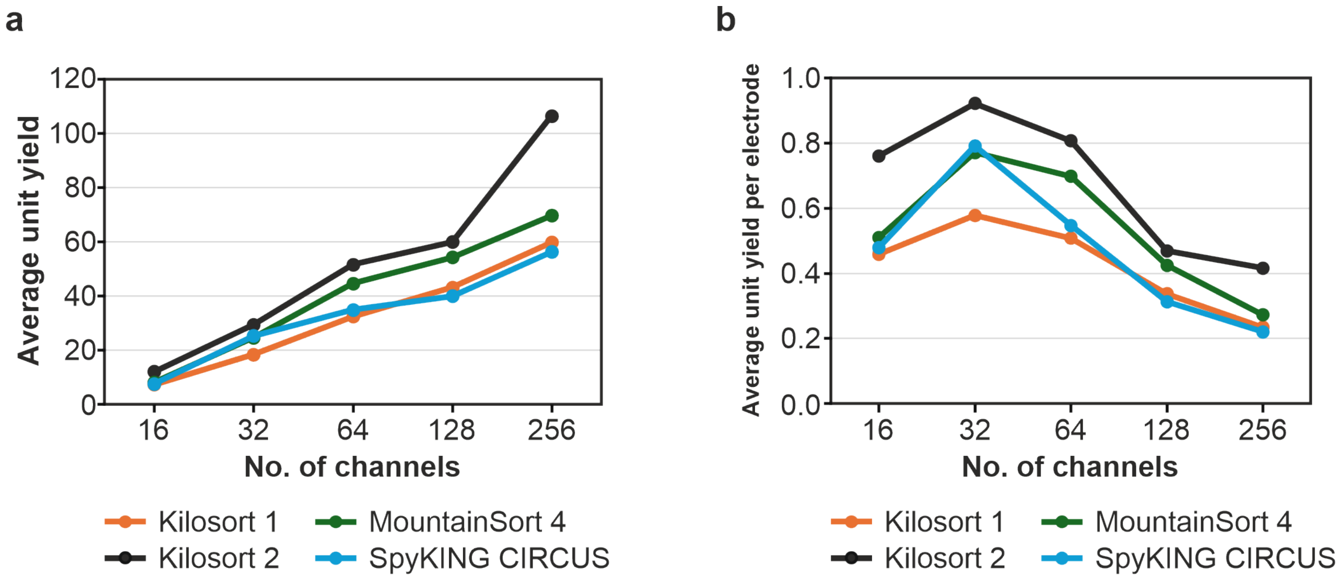

Supplementary Figure 6. (**a**) Average number of single units identified in the rat neocortical recordings with different spike sorting algorithms. (**b**) The single unit yield divided by the number of electrodes.
